# Physical Exercises from a National Standpoint

**Published:** 1917-07-07

**Authors:** 


### PHYSICAL EXERCISES FROM A NATIONAL STANDPOINT.

Bodily fitness just now occupies a prominent position in the public attention, and this for obvious reasons. The young manhood of the nation is exercised in a supreme struggle, where strength and agility and power of endurance are the conditions of victory. The tests are severe, and the result, considered broadly, does not reflect on the national vigour. Not only from the battlefield, but also from the stress of our industrial centres, comes the verdict that the race has lost none of its traditional stamina. Such a conclusion would, however, be qualified in reference to many individuals were the records of our recruiting stations and training camps made public. And one of our most careful social students has recently published a severe condemnation of the great mass of our people as judged by a moderate standard of manly vigour, graceful bearing, and good looks. The gloomy stories of national degeneration which were so popular a few years ago have obviously suffered the contradiction of events, but this by no means implies that the question may safely be pushed aside. There is, indeed, good reason for emphasising the proposition advanced by Sir William Milligan in a recent lecture, that physical efficiency is one of our greatest national assets.

In its wide and general application this proposition may safely challenge contradiction. The truth is vivid even to the most sluggish mind. But the doctrine is less generally recognised when it is applied to the individual. Yet here also it is emphatically true. There are exceptions no doubt, but, granting these, personal efficiency not only in bodily but also in mental work is promoted by bodily fitness; and equally a sense of well-being, of capacity, and of the joy of living depends on its possession. Even the smooth running of domestic and other machinery does not escape its influence. It is therefore an aim to be attempted, and we are quite prepared to admit that greater attention to the subject would be a profitable exercise. The test applies more particularly to the industrial classes, where the children have none of the stimulus of sport and games provided by the public schools and the universities. Moreover, these children are plunged into the serious business of wage-earning at an age when their more fortunate contemporaries have yet some years of educational discipline to compass. Sir William Milligan's remedy is compulsory drill for two hours a week in all primary and secondary schools, and carried on until the child is eighteen years of age. He makes the excellent suggestion that such drill should be conducted in the open spaces of our public parks rather than in the school playgrounds, where the surroundings are usually anything but cheerful. It is not improbable that some fashion of drill and training will be imposed on the nation as one of the educational lessons of the war. But drill even in its best forms lacks the swift mental stimulus and sense of personal keenness which are important elements in the development of the sound body. There must be interest as well as exercise if the youth of the new generation are to acquire in greater measure than their forbears the quality of bodily efficiency. Not submission, but a sense of achievement, is the atmosphere in which the fullest physical training is accomplished.

